# *Bacillus cereus* Typhlitis in a Patient with Acute Myelogenous Leukemia: A Case Report and Review of the Literature

**DOI:** 10.1155/2018/7510715

**Published:** 2018-03-11

**Authors:** James D. Denham, Sowmya Nanjappa, John N. Greene

**Affiliations:** ^1^Morsani College of Medicine, University of South Florida, Tampa, FL, USA; ^2^H. Lee Moffitt Cancer Center, Tampa, FL, USA

## Abstract

*Bacillus cereus* is a Gram-positive rod that is now recognized as a rare cause of frank disease in the neutropenic hematologic malignancy patient. Because this pathogen is rarely isolated in clinical specimens, no large studies exist to guide the management of these acutely ill patients. Individual case reports and case series exist in the literature describing various clinical manifestations of *B. cereus* in the neutropenic patient including bacteremia/septicemia, pneumonia, meningitis/encephalitis, hepatic abscesses, and gastritis. In this report, we describe a case of typhlitis caused by *B. cereus* in a 74-year-old female with recently diagnosed acute myelogenous leukemia (AML), and we summarize the available English language literature to draw tentative conclusions regarding the clinical manifestations of this organism.

## 1. Background

*Bacillus cereus* is a Gram-positive, spore-forming rod that classically causes food poisoning via plasmid-encoded toxins. Life-threatening infections with *B. cereus* have been reported in patients with hematologic malignancies, primarily as these patients experience neutropenia following the initiation of induction chemotherapy. Here, we report a case of *B. cereus* colitis/typhlitis in a neutropenic patient with acute myelogenous leukemia (AML), and we summarize the literature regarding the infectious manifestations of *B. cereus* in patients with hematologic malignancies. This is the second reported case of *B. cereus* typhlitis.

## 2. Case Report

A 74-year-old Caucasian female with a 2-month history of pancytopenia and a preliminary diagnosis of myelodysplastic syndrome (MDS) presented to our tertiary cancer treatment facility for further evaluation. A repeat bone marrow biopsy was performed and demonstrated 26% blasts, and the patient was formally diagnosed with acute myelogenous leukemia (AML). One month later, the patient was admitted to our facility for induction “7 + 3” chemotherapy with sorafenib. On admission, the patient was found to have mild pancytopenia with an absolute neutrophil count (ANC) between 500 and 1,000 cells/*μ*L but had no specific complaints and an unremarkable physical examination.

Following initiation of chemotherapy, antimicrobial prophylaxis with ciprofloxacin, acyclovir, and micafungin was begun, and nebulized amphotericin B was added because computed tomography (CT) scan of the chest demonstrated several ground-glass infiltrates with no pulmonary symptoms. The ground-glass infiltrates remained stable on follow-up CT scan. Mild hemoptysis developed and empiric cefepime was begun in place of oral ciprofloxacin on the 8th day of neutropenia due to fever. She remained stable and well appearing until the 10th day of neutropenia when nonbloody diarrhea without abdominal pain developed. *Clostridium difficile* stool polymerase chain reaction (PCR) analysis was negative. Loperamide was administered to help reduce the diarrhea.

Diarrhea persisted despite escalating doses of loperamide, and on the 13th day of neutropenia, right upper quadrant abdominal pain developed and metronidazole 500 mg twice daily was initiated. CT scan of the abdomen and pelvis revealed cecal wall thickening associated with fat stranding and right paracolic gutter fluid collection—findings consistent with typhlitis. Labs revealed an ANC of less than 500 cells/*μ*L. Stool cultures obtained the following day revealed a preponderance of *Bacillus cereus* with the absence of usual enteric flora. Physical examination then demonstrated dull periumbilical pain upon palpation and a sharper pain in the right lower quadrant of the abdomen. Metronidazole was changed to clindamycin 450 mg thrice daily. During the next several days, the abdominal pain and diarrhea resolved. The patient's later hospital course was complicated by vancomycin-resistant *Enterococcus* (VRE) bacteremia that developed after 21 days of neutropenia and was treated with central venous catheter removal and linezolid 600 mg twice per day. She would finish the 2-week linezolid course outpatient; she was discharged afebrile after 30 hospital days with an ANC of 3.28 cells/*μ*L. Complete clinical remission of the AML was ultimately achieved following 2 rounds of consolidation chemotherapy.

## 3. Discussion

*Typhlitis*, also known as *neutropenic enterocolitis* (or, rarely, *ileocecal syndrome*) is the most common cause of fever and abdominal tenderness in the neutropenic patient [1]. Historically, *Clostridium septicum* was pointed to as a common etiologic agent of typhlitis [2]. It is known, however, that the microbiologic etiology of this disease is broad and diverse, with an 18-year study in leukemic children finding that 84% and 16% of cases being caused by bacteria and fungi, respectively [3]. Currently, there exist no high-quality case-control, cohort, or randomized controlled trials to guide clinicians in the best management of typhlitis [4].

The isolation of *B. cereus* from clinical specimens was historically considered contamination. However, it is now clear from the literature that *B. cereus* can cause a wide spectrum of disease in the neutropenic patient. Our patient, a 74-year-old female undergoing induction chemotherapy for AML, was found to have typhlitis with stool culture confirming the presence of *B. cereus* in the colon with a notable absence of the usual enteric flora. The initiation of clindamycin led to a rapid improvement in our patient's symptoms. Our patient's blood cultures remained sterile during the episode of typhlitis.

On review of the literature, we found a handful of reports describing the various clinical manifestations of *B. cereus* (Table 1). The most common manifestation was sepsis. As reported by Uchino et al., patients who develop central nervous system (CNS) symptoms or who have bacterial involvement of the liver tend to have a worse prognosis [5, 9]. Indeed, from our review, it is clear that while *B. cereus* bacteremia can result in the seeding of almost any organ, *B. cereus* seems to preferentially seed the brain/meninges and the liver [5, 7, 9–12]. CNS involvement is most commonly multifocal and leads to a rapid deterioration in mental status and frequently leads to development of a comatose state. Liver involvement is often asymptomatic but can manifest on CT scan as multiple subcentimeter, hypodense lesions. Pathologic analysis of the liver at autopsy reveals microabscesses with large Gram-positive rods present within the lesions [10, 12].

The management of the neutropenic patient with cultures positive for *B. cereus* presents a clinical dilemma as no large studies exist to guide the management of these critically ill patients. From our review of the available English language literature, some tentative conclusions may be drawn regarding this organism in the context of the neutropenic hematologic malignancy patient. These conclusions are summarized in Table 2.

## Figures and Tables

**Table 1:** Summary of literature review findings.

Reference Number	Author	Summary
[5]	Uchino et al.	This article reports 13 cases of septicemia caused by *B. cereus*. Uchino et al. report a case fatality rate of 25% and found that the presence of liver and CNS involvement portended a poorer prognosis. Clindamycin resistance was found to be high in this series (76.9%), and Uchino et al. recommend against using clindamycin and carbapenems in the empiric treatment of *B. cereus* infections.
[6]	Frankard et al.	This article reports a case of pneumonia caused by *B. cereus* in the context of a neutropenic patient with ALL. This patient was treated with VCN and demonstrated clinical improvement. She was readmitted 3 weeks later with a recurrent pneumonia due to *Streptococcus* spp., and she expired due to this later infection.
[7]	Marley et al.	Marley et al. report a case of meningoencephalitis occurring in a patient undergoing induction chemotherapy for AML. The patient became febrile with an ANC of 20/mm^3^. Over several hours, the patient experienced a rapid deterioration of mentation, and a CT scan revealed multiple enhancing lesions in the brain. The patient died 12 hours following the onset of neurological symptoms. Autopsy additionally found multiple liver abscesses.
[8]	Le Scanff et al.	Le Scanff et al. report a case of necrotizing gastritis in a female patient with AML 63 days following induction chemotherapy initiation. The patient developed severe epigastric pain and massive hematemesis leading to hemodynamic instability; blood and gastric mucosal cultures revealed *B. cereus*. This patient was successfully treated with combination of VCN and imipenem.
[9]	Inoue et al.	This substantial article reports 23 cases of *B. cereus* bacteremia in HM patients, with 12 of these patients developing a frank sepsis. Inoue reports a case fatality rate of 25% for *B. cereus* sepsis. Further, independent risk-factor analysis concluded that a low ANC, CV catheterization, and CNS symptoms were all significantly associated with a poorer prognosis.
[10]	Ginsburg et al.	Ginsburg et al. report a fatal case of *B. cereus* sepsis that arose in a 22-year-old male following induction chemotherapy for AML. On hospital day 5, the patient became febrile and neutropenic and a CT scan revealed findings consistent with colitis, but stool *C. difficile* testing remained negative. His symptoms resolved by hospital day 14 with metronidazole and imipenem. However, 6 days later, he developed diffuse abdominal pain and blood cultures revealed *B. cereus*. VCN and ampicillin were added, but a new CT revealed pancolitis and multiple hypodense lesions in the liver. The patient expired on the thirty-fourth hospital day. Autopsy confirmed the presence of *B. cereus* in the liver microabscesses.
[11]	Musa et al.	This case series describes 3 patients, 2 with AML and 1 with ALL, who develop septicemia due to *B. cereus*. All 3 patients died despite treatment with amikacin. Additionally, all 3 patients developed a similar syndrome of abnormal posturing and clinical signs of brain stem death. 1 patient in this case series was found to have elevated liver enzymes and serum bilirubin perimortem.
[12]	Akiyama et al.	Akiyama et al. report a case of fatal *B. cereus* septicemia in a 64-year-old patient with AML undergoing induction chemotherapy. Autopsy revealed a necrotizing infection in the leptomeninges of the brain and spinal cord and numerous microabscesses of the liver. *B. cereus* was present within these lesions.

CNS = central nervous system; ALL = acute lymphoblastic leukemia; VCN = vancomycin; AML = acute myelogenous leukemia. ANC = absolute neutrophil count. CT = computed tomography. HM = hematologic malignancy. CV = central venous. *C. difficile* = *Clostridium difficile*.

**Table 2:** Conclusions for *Bacillus cereus* infection in neutropenic hematologic malignancy patients.

	Conclusion	Reference Number
1	Neutropenia is the primary risk factor for *infection*, as opposed to *intoxication*, with *B. cereus*	[5–12]
2	*B. cereus* has a predilection for the CNS and the liver; involvement of either of these two organ systems portends a poor prognosis	[5, 7, 9–12]
3	The case fatality rate for *B. cereus* sepsis appears to be 25%	[5, 9]
4	The most commonly used definitive therapy for *B. cereus* infection appears to be vancomycin with or without carbapenem	[5–10]

CNS = central nervous system.
